# 2600. Incidence of Non-COVID Pneumonia Before and After Mask Mandate at a Midwestern Hospital: An Analytical Cross-Sectional Study

**DOI:** 10.1093/ofid/ofad500.2215

**Published:** 2023-11-27

**Authors:** Stephen B Szabadi, Katelyn Koenig, Chester Gauss, Zachary Sila, Adithya Reddy, Cuyler Huffman, John Dewey, Gordon Liu, Melissa H Olken

**Affiliations:** Western Michigan Homer Stryker MD School of Medicine, Kalamazoo, Michigan; Western Michigan Homer Stryker MD School of Medicine, Kalamazoo, Michigan; Western Michigan Homer Stryker MD School of Medicine, Kalamazoo, Michigan; Western Michigan Homer Stryker MD School of Medicine, Kalamazoo, Michigan; Western Michigan Homer Stryker MD School of Medicine, Kalamazoo, Michigan; Western Michigan Homer Stryker MD School of Medicine, Kalamazoo, Michigan; West Virginia University School of Medicine, Morgantown, West Virginia; Spectrum Health, Grand Rapids, West Virginia; Western Michigan Homer Stryker MD School of Medicine, Kalamazoo, Michigan

## Abstract

**Background:**

The introduction of universal masking policies at many American healthcare institutions during the COVID-19 pandemic represented a major shift in infection prevention protocol. However, despite their widespread use, previous data on face masks as a tool for preventing respiratory infections before COVID have been mixed, frequently showing no benefit. This study examines the impact of a universal face mask policy on rates of hospital-acquired pneumonia (HAP), ventilator-associated pneumonia (VAP), community-acquired pneumonia (CAP), and other non-COVID pneumonias of indeterminate etiology at Ascension Borgess Hospital in Kalamazoo, Michigan.

**Methods:**

An analytical cross-sectional study was performed comparing patient data from March through September 2019 with the same period in 2020 for any patient admitted with any ICD-10 code for pneumonia. Chi-square analysis was employed to determine pneumonia frequency by subtype, pre and post-mandate. A significance level of *p* < 0.05 was used for all tests.

**Results:**

1187 patient records were collected, 28 were excluded for incompleteness, and 1159 were included in our analysis. The study population was 81.80% White and 53.66% male. 670 patients (57.81%) were pre-mask mandate and 487 (42.19%) were post-mandate. Pre-mandate, 107 patients had HAP, 575 had all-cause pneumonia (non-COVID pneumonia of any etiology), and 0 had VAP. Post-mandate, 84 patients had HAP, 2 had VAP, and 376 had all-cause pneumonia. A significant difference was observed in the number of all-cause pneumonia cases between pre and post-mandate (χ^2^ = 13.3058, p < 0.0001), but not in HAP cases (χ2 = 0.2996, p = 0.5841). There were only two instances of VAP; therefore, inferential statistics were not obtained.

**Figure:**
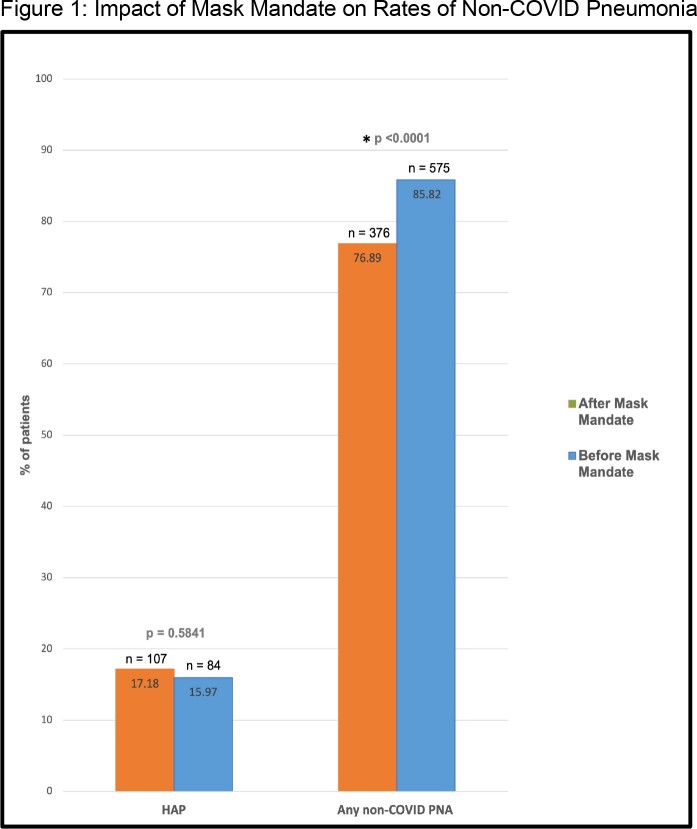

A significant difference was observed in all-cause pneumonia cases between pre and post-mask mandate implementation (χ2 = 13.3058, p <0.0001), but not in HAP cases (χ2 = 0.2996, p = 0.5841).

**Conclusion:**

Among hospitalized patients, a significant difference in HAP pre and post-mandate was not observed. However, the incidence of non-COVID pneumonia of any etiology was significantly lower during the mask mandate (p < 0.0001). Wether this difference is attributable to universal masking or other mandated or non-mandated precautionary behaviors in the hospital or community cannot be determined. These results suggest that mask mandates as currently implemented do not prevent HAP, but may play a role in decreasing total pneumonia prevalence in the community.

**Disclosures:**

**All Authors**: No reported disclosures

